# Supplementary material for: Deciphering brain organoid heterogeneity by identifying key quality determinants
Source: Commun Biol. 2025 Oct 1;8:1412. doi: 10.1038/s42003-025-08855-6 (PMC12488937; doi:10.1038/s42003-025-08855-6)
Supplement: Supplementary file 1 — Supplementary Information [file 42003_2025_8855_MOESM1_ESM.pdf]

Mesenchymal Cell Proportion and Morphology Define the Quality of Brain Organoids

Supplementary files

Supplementary Table S1. Cell lines used in the study

Cell line	Short naming	Cell type	Gender	Passage	
H9	H9	hES	Female	p41	
HuES6	HuES6	hES	Female	p35	
KOLF2.1J	KOLF	iPSC	Male	p+4*	* - passage from the moment the line was obtained
WTSIi013-A	Kucg2	iPSC	Male	p38	
HPSI0314i-sojd_3	Sojd3	iPSC	Female	p33	
CV-hIPS-B	CVB	iPSC	Male	p38	
TMOi001A	Thermo	iPSC	Female	p26	
UKERi4CC-S1-015	4CC	iPSC	Male	p16	
UKERiRN4-S1-009	RN4	iPSC	Female	p12	
UKERi4L6-S1-027	4L6	iPSC	Male	p20	
UKERi33Q-S1-101	33Q	iPSC	Female	p12	
UKERi82A-S1-002	82A	iPSC	Female	p15	

Supplementary Table S2. Youden's J Statistics for brain organoid morphological parameters

Parameter	Optimal cutoff	Youden's Index	Sensitivity (%)	Specificity (%)	Description
**Area**	5695105.348	0.67	77%	88.9%	The total number of pixels enclosed within the object boundary (in μm^2 or $pixels^2$).
**Perimeter**	13400.056	0.65	96.3%	68.9%	The length of the boundary of the object (in μm or pixels).
**Circularity**	0.298	0.52	77.8%	74.1%	Ratio of the major axis to the minor axis of the fitted ellipse (Major/Minor).
**Feret**	3050.356	0.68	92.6%	75.6%	The longest distance between any two points along the object boundary.
**Aspect Ratio**	1.217	0.33	77.8%	55.6%	Calculated as 4 × Area / (π × $Major^2$); value of 1 indicates a perfect circle.
**Roundness**	0.824	0.33	55.6%	77.8%	Calculated as 4π × Area / ($Perimeter^2$); ranges from 0 (line) to 1 (circle).
**Solidity**	0.909	0.53	82.2%	70.4%	Ratio of the Area to the Convex Hull Area; reflects how concave the object is.
**Cysts.Area**	0.0895	0.67	96.3%	71.1%	Ratio of Area with cysts / total organoid Area
**Cysts.Amount**	2	0.53	81.5%	71.1%	Number of cysts in one organoid

Supplementary Figure S1. The k-means elbow method is used to determine the optimal number of clusters

A

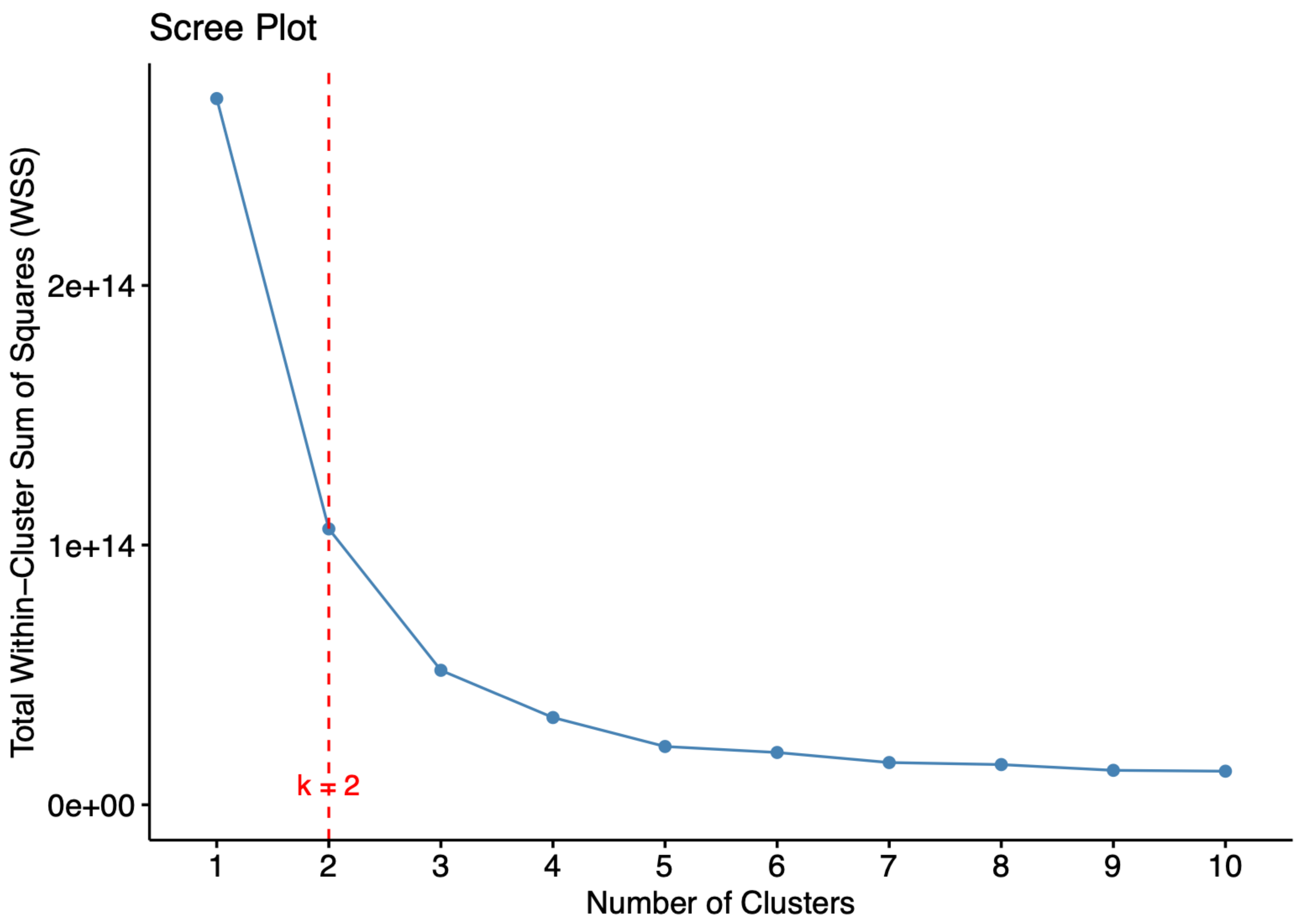

Supplementary Figure S1. The k-means elbow method is used to determine the optimal number of clusters. The x-axis represents the number of clusters, ranging from 1 to 10. The y-axis displays the Total Within-Cluster Sum of Squares (WSS). A red dashed vertical line marks the position where k equals 2 - the suggested optimal number of clusters based on the elbow method.

Supplementary Figure S2. Morphology-based PCA plot of day 30 (d30) brain organoids.

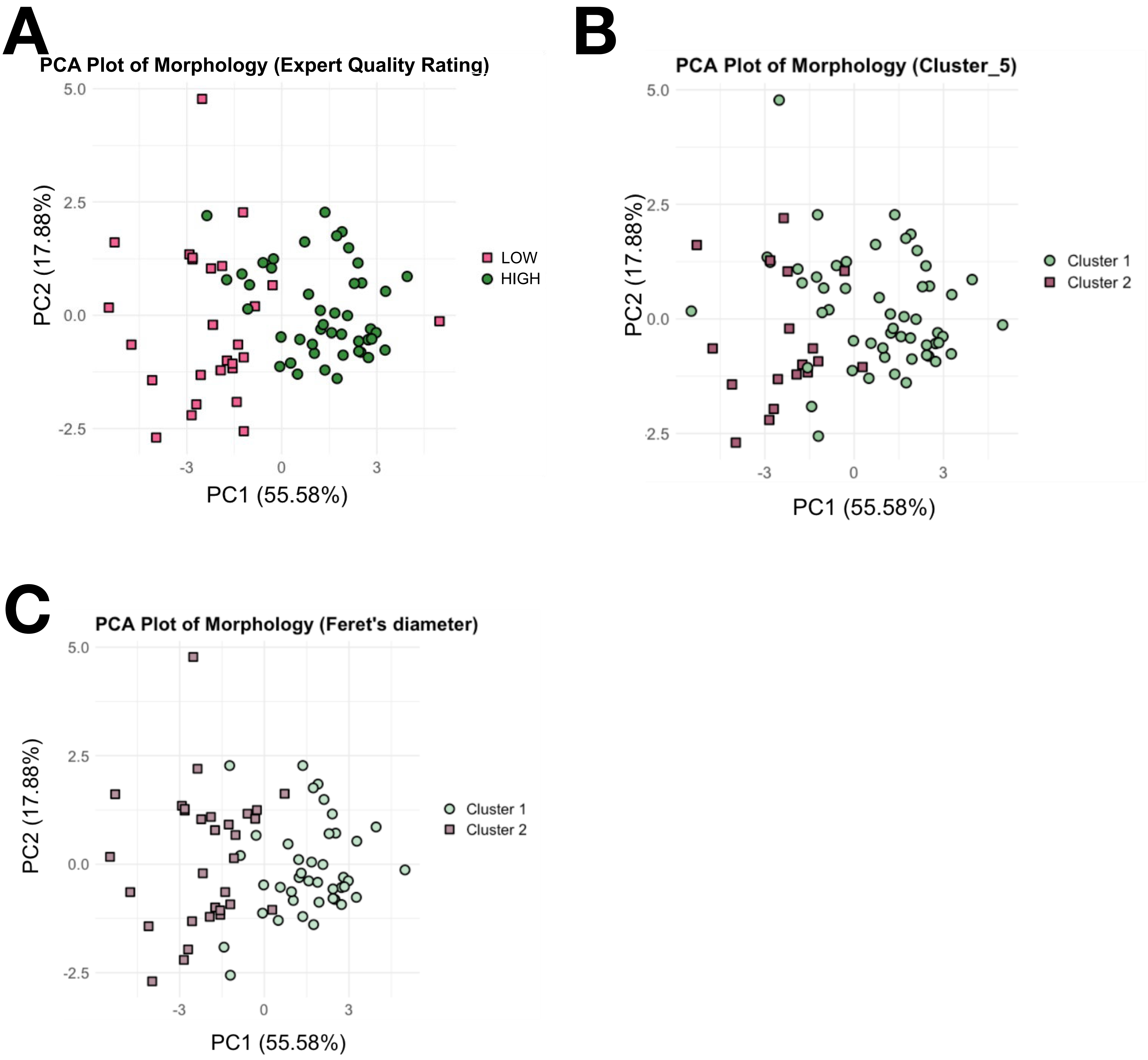

Supplementary Figure S2. Morphology-based PCA plot of day 30 (d30) brain organoids.
Principal Component Analysis (PCA) plot illustrating the morphology of d30 brain organoids, with samples color-coded according to various quality assessment methods. **A.** Samples are color-coded based on Expert evaluation of organoid quality: green represents high-quality organoids, and pink represents low-quality organoids. **B.** Samples are color-coded according to k-means clustering results, using five morphological parameters (Feret, Area, Cyst Amount, Cyst Area, and Perimeter). Cluster 1 corresponds to the high-quality organoid group, while Cluster 2 aligns with the low-quality organoid group, as per the expert evaluation. **C.** Samples are color-coded based on Youden's J statistic for the optimal cutoff of Feret diameter. Cluster 1 represents organoids classified as high-quality (less than 3050 μm in diameter), and Cluster 2 represents organoids classified as low-quality (more than 3050 μm in diameter), consistent with expert evaluation.
The variance explained by Principal Component 1 (PC1) is 55.58%, and by Principal Component 2 (PC2) is 17.88%.

Supplementary Figure S3. Schematic representation of Feret diameter

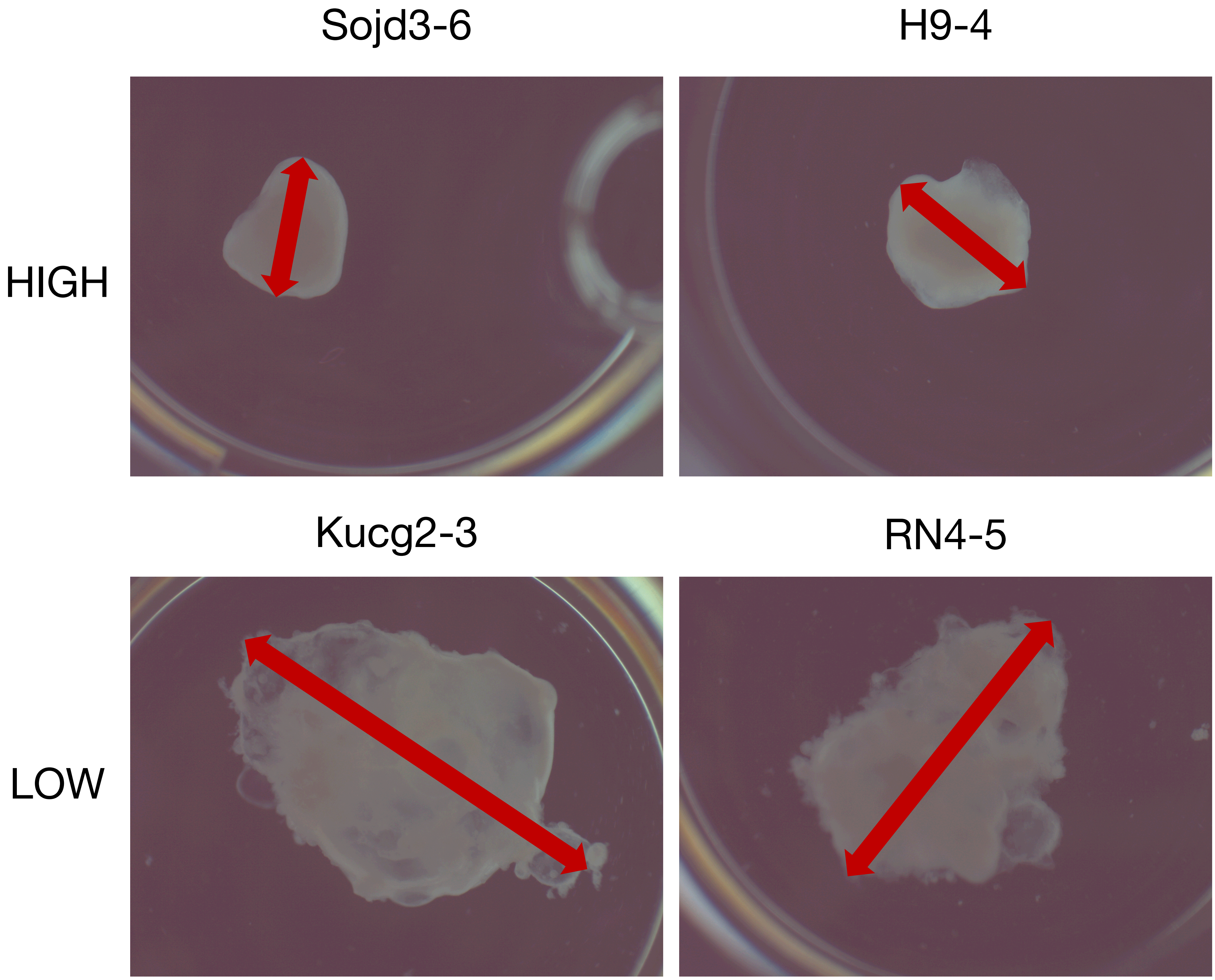

Supplementary Figure S3. Schematic representation of Feret diameter
4 representative organoid images used for analysis of high-quality and low-quality organoids in 4 different hPSC-lines. Schematic Feret diameter as red arrow. Low-quality organoids exhibit a tendency for a higher Ferret diameter.

Supplementary Figure S4. WebCSEA - Web-based Cell-type Specific Enrichment Analysis of Genes for Cell type determination

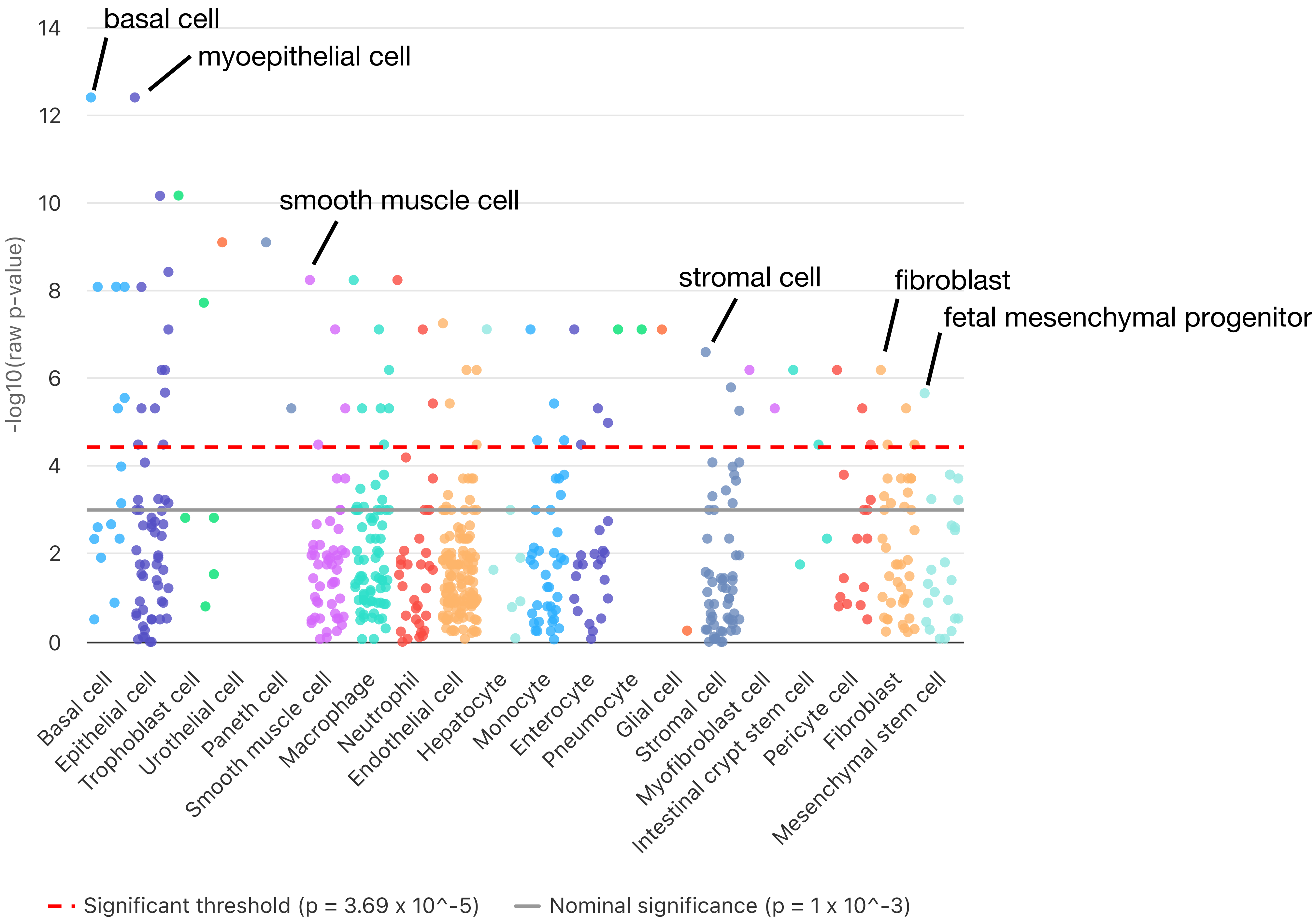

Supplementary Figure S4. WebCSEA - Web-based Cell-type Specific Enrichment Analysis of Genes for Cell Type Determination. Jitter plots of –log10 raw (left) P-values among 1355 tissue-cell types by top 20 enriched general cell types, defined by the WebCSEA, based on the list of common differentially expressed genes from the three brain organoid evaluation approaches. The red dashed line indicates the Bonferroni-corrected significance (P = 3.69 × 10–5) by 1355 tissue-cell types. The grey solid line indicates the nominal significance (P = 1 × 10–3).

Supplementary Figure S5. Correlation of Feret diameter and predicted mesenchymal cell content with gene expression of ENG and NT5E

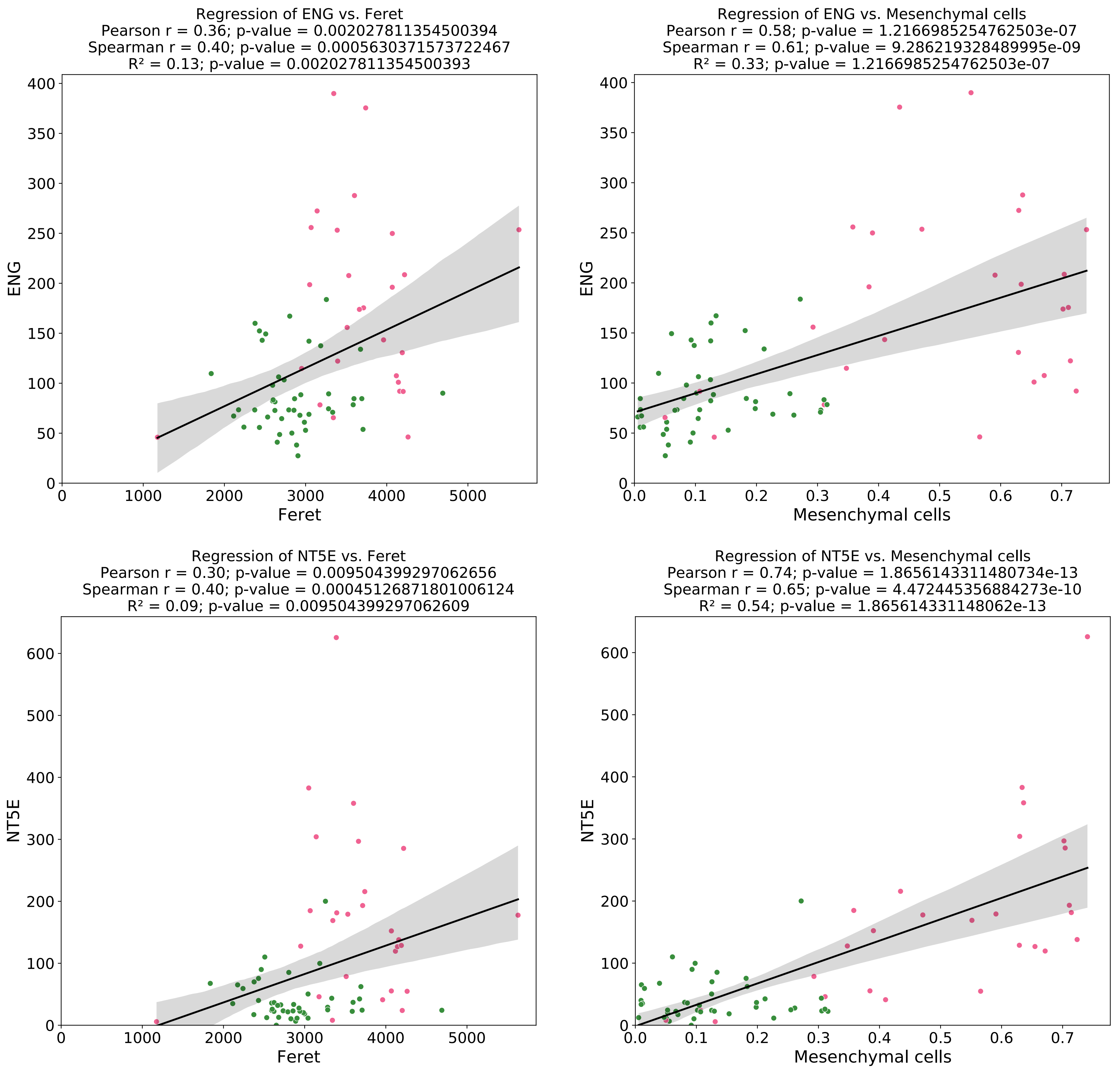

Supplementary Figure S5. Correlation of Feret diameter and predicted mesenchymal cell content with gene expression of ENG and NT5E. Scatter plots correlating Feret diameter (left plots) or predicted mesenchymal cell content (right) from RNA-seq deconvolution with gene expression (DESeq2 normalized counts) of mesenchymal cell associated genes ENG (CD105), NT5E (CD73). Individual organoid classification by expert depicted in red dots (LOW) and green dots (HIGH). Correlation coefficient (Pearson and Spearman) and regression coefficient with respective P values on top of each graph.

Supplementary Figure S6. CD105 and CD73 intensity in organoids based on PSC-line organoid majority quality rating

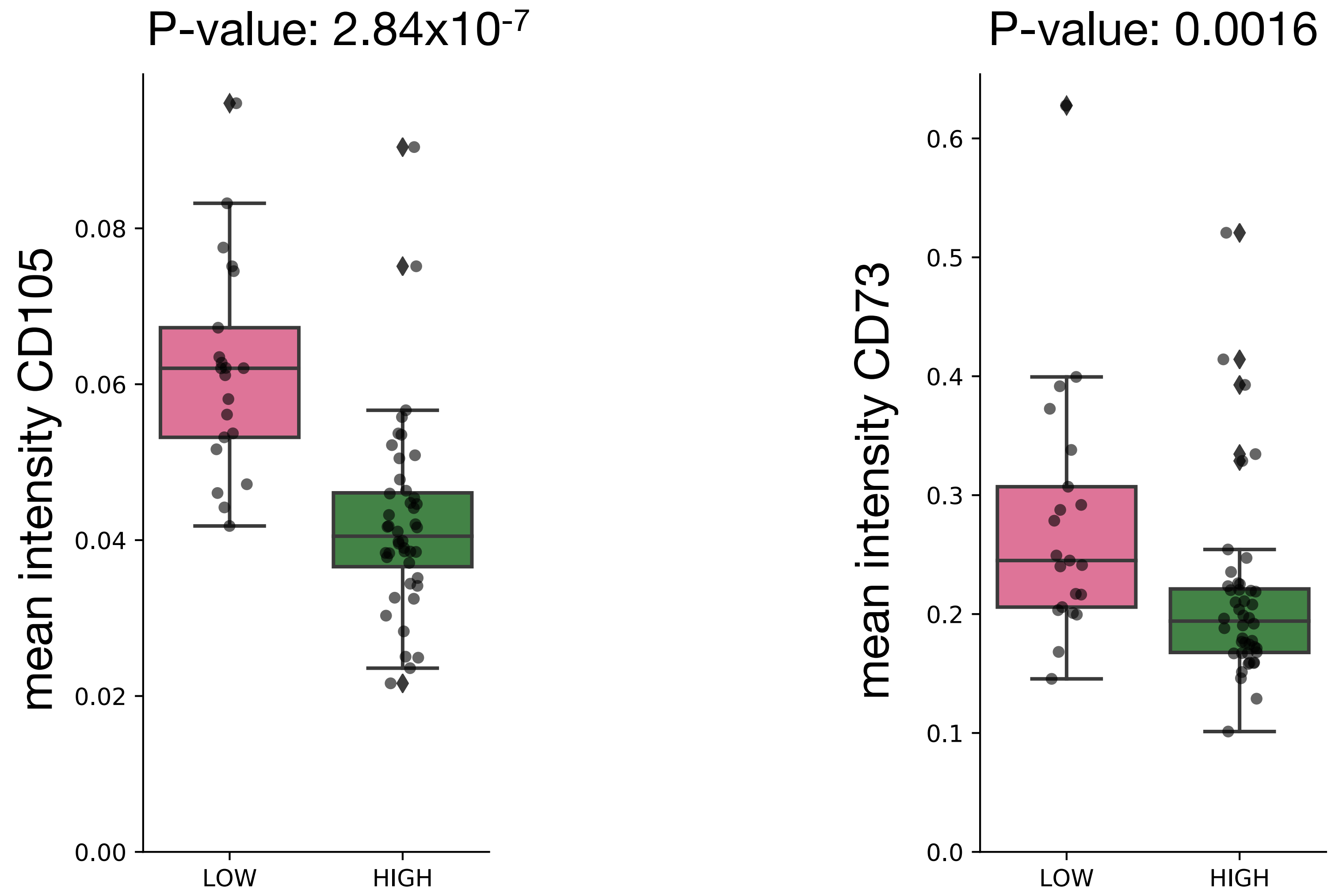

Supplementary Figure S6. CD105 and CD73 intensity in organoids based on PSC-line organoid majority quality rating. The 12 PSC lines used in the study were separated in two groups (majority as high or low quality). Fluorescence intensity of CD105 (left) or CD73 (right) was quanified. Graphs are box plots with median as central line. P-values depicted on top (Rank-sum test). Individual organoids depicted as dots.
