# Supplementary material for: Deciphering brain organoid heterogeneity by identifying key quality determinants
Source: Commun Biol. 2025 Oct 1;8:1412. doi: 10.1038/s42003-025-08855-6 (PMC12488937; doi:10.1038/s42003-025-08855-6)
Supplement: Supplementary file 4 — Reporting Summary [file 42003_2025_8855_MOESM4_ESM.pdf]

Corresponding author(s): Floran Krach MD/PhD

Last updated by author(s): Jun 28, 2025

Reporting Summary

Nature Portfolio wishes to improve the reproducibility of the work that we publish. This form provides structure for consistency and transparency in reporting. For further information on Nature Portfolio policies, see our Editorial Policies and the Editorial Policy Checklist.

Statistics

For all statistical analyses, confirm that the following items are present in the figure legend, table legend, main text, or Methods section.

n/a	Confirmed	
☐	☒	The exact sample size (n) for each experimental group/condition, given as a discrete number and unit of measurement
☐	☒	A statement on whether measurements were taken from distinct samples or whether the same sample was measured repeatedly
☐	☒	The statistical test(s) used AND whether they are one- or two-sided<br>*Only common tests should be described solely by name; describe more complex techniques in the Methods section.*
☐	☒	A description of all covariates tested
☐	☒	A description of any assumptions or corrections, such as tests of normality and adjustment for multiple comparisons
☐	☒	A full description of the statistical parameters including central tendency (e.g. means) or other basic estimates (e.g. regression coefficient) AND variation (e.g. standard deviation) or associated estimates of uncertainty (e.g. confidence intervals)
☐	☒	For null hypothesis testing, the test statistic (e.g. F, t, r) with confidence intervals, effect sizes, degrees of freedom and P value noted<br>*Give P values as exact values whenever suitable.*
☐	☒	For Bayesian analysis, information on the choice of priors and Markov chain Monte Carlo settings
☐	☒	For hierarchical and complex designs, identification of the appropriate level for tests and full reporting of outcomes
☐	☒	Estimates of effect sizes (e.g. Cohen's d, Pearson's r), indicating how they were calculated

Our web collection on statistics for biologists contains articles on many of the points above.

Software and code

Policy information about availability of computer code

Data collection	flow cytometry data collection: CytExpert, Beckman Coulter, https://www.beckman.com/flow-cytometry/instruments/cytoflex/software
Data analysis	CytExpert (version 2.4), Beckman Coulter, https://www.beckman.com/flow-cytometry/instruments/cytoflex/software;<br>ZEN blue (version 2.6), Zeiss, https://www.zeiss.com/microscopy/us/products/microscope-software.html;<br>Prism (ver. 10.2.0), GraphPad Software, Inc. http://graphpad.com/scientific-software/prism;<br>Python 3.0;<br>seaborn package;<br>scipy.stats package;<br>R (version 4.0.2)<br>tidyverse (version 2.0.0);<br>factoextra (version 1.0.7);<br>STAR (version 2.7.9a), Dobin et al., 2013 https://github.com/alexdobin/STAR;<br>Samtools (version 1.9.0), Li et al, 2009, http://samtools.sourceforge.net/;<br>featureCounts tool as part of the Rsubread package (ver. 2.16.1);<br>DESeq2 R package (ver. 1.42.1);<br>org.Hs.eg.db annotation package (ver. 3.18.0);<br>clusterprofiler (ver. 4.10.1);<br>enrichplot (ver. 1.22.0);<br>pROC (ver. 1.18.5)<br>cutpointr (ver. 1.1.2);<br>eulerr (ver. 7.0.2);;

RStudio software (ver. 2023.09.1+494);
BayesPrism (ver. 2.0;
Human Neural Organoid Cell Atlas;
WebCSEA;

For manuscripts utilizing custom algorithms or software that are central to the research but not yet described in published literature, software must be made available to editors and reviewers. We strongly encourage code deposition in a community repository (e.g. GitHub). See the Nature Portfolio guidelines for submitting code & software for further information.

Data

Policy information about availability of data

All manuscripts must include a data availability statement. This statement should provide the following information, where applicable:

- Accession codes, unique identifiers, or web links for publicly available datasets
- A description of any restrictions on data availability
- For clinical datasets or third party data, please ensure that the statement adheres to our policy

Data has been deposited at the European Genome-phenome Archive (EGA), which is hosted by the EBI and the CRG, under accession number EGAS50000000659. Further information about EGA can be found at https://ega-archive.org and "The European Genome-phenome Archive of human data consented for biomedical research.

Research involving human participants, their data, or biological material

Policy information about studies with human participants or human data. See also policy information about sex, gender (identity/presentation), and sexual orientation and race, ethnicity and racism.

Reporting on sex and gender	Information in regards to sex was available for all PSC lines used and reported in Supplementary Table S1. Information in regards to gender is not available. No sex or gender analyses were performed.
Reporting on race, ethnicity, or other socially relevant groupings	not applicable
Population characteristics	To adjust the differences between different cell lines, the design formula included Cell lines as a covariate (design: ~ Condition + Cell_lines) in the RNA-seq analysis.
Recruitment	No specific recruitment was performed for this study. All cell lines were present on-site.
Ethics oversight	The human cells used in this study were handled in accordance with the principles outlined in the Declaration of Helsinki. Concerning all UKER lines, informed written consent was obtained from the participating individuals to use donor tissue for research purposes. The generation and use of local human iPSC lines were approved by the Institutional Review Board of the University Hospital of Erlangen (Nr. 4120 and 259_17B: Generation of human neuronal models for neurodegenerative diseases). Concerning iPSC lines Kucg2 and Sojd3 (HipSci feeder-free panel (ECACC 77659901)), the MTA was obtained from the Wellcome Trust Sanger Institute for research purposes. The remaining iPSC lines (TMOi001A, KOLF2.1, CVB) are commercially available. The use of human embryonic stem cells for this project was approved by the Central Ethics Committee for Stem Cell Research (121. Approval according to the German Stem Cell Act to Beate Winner).

Note that full information on the approval of the study protocol must also be provided in the manuscript.

Field-specific reporting

Please select the one below that is the best fit for your research. If you are not sure, read the appropriate sections before making your selection.

☒ Life sciences ☐ Behavioural & social sciences ☐ Ecological, evolutionary & environmental sciences

For a reference copy of the document with all sections, see nature.com/documents/nr-reporting-summary-flat.pdf

Life sciences study design

All studies must disclose on these points even when the disclosure is negative.

Sample size	No statistical methods were used to determine the sample size in this study. 12 hPSC lines from different donor
Data exclusions	No data was excluded
Replication	All data are gathered from one parallel culture / differentiation round of all available cell lines. The individual fibroblast lines / iPSC clones used for each experiment represent our replicates.
Randomization	For the in vitro studies, we did not perform any randomization. To reduce variability, all data points depicted in a graph resulted from an experiment where all lines were processed in parallel, including cell culture, reagent batch, sample preparation and analysis.

Blinding	Blinding was not performed for in vitro studies since the investigator was aware of the disease status of each of the lines used.

Reporting for specific materials, systems and methods

We require information from authors about some types of materials, experimental systems and methods used in many studies. Here, indicate whether each material, system or method listed is relevant to your study. If you are not sure if a list item applies to your research, read the appropriate section before selecting a response.

Materials & experimental systems

n/a	Involved in the study
☐	☒ Antibodies
☐	☒ Eukaryotic cell lines
☒	☐ Palaeontology and archaeology
☒	☐ Animals and other organisms
☒	☐ Clinical data
☒	☐ Dual use research of concern
☒	☐ Plants

Methods

n/a	Involved in the study
☒	☐ ChIP-seq
☐	☒ Flow cytometry
☒	☐ MRI-based neuroimaging

Antibodies

Antibodies used	SOX2 (3579S, Cell Signaling Technology); MAP2 (M9942, Sigma-Aldrich); COL1A1 (3G3): 1:100 (sc-293182, Santa Cruz)).)), CD73 (ab317364, abcam), CD105 (ab252345, abcam), -PAX6-APC (1:50, Miltenyi Biotec, 130-123-267); anti-MAP2-PE (1:100, Merck, FCMAB318PE)
Validation	All antibodies were validated by vendors.

Eukaryotic cell lines

Policy information about cell lines and Sex and Gender in Research

Cell line source(s)	H9 (hES, female, WiCell Research Institute - National Stem Cell Bank), HuES6 (hES, female, Harvard University), KOLF2.1 (hiPSC, male, Cold Spring Harbour), WTSli013-A (hiPSC, male, EBiSC banking facility is located at the Babraham Research Campus), HPSI0314i-sjod_3 (hiPSC, female, Culture Collections UK Health Security Agency) CV-hiPS-B (hiPSC, male, Coriell Institute for Medical Research), TMOi001A (hiPSC. female, Thermo Fisher Scientific), UKERi4CC-S1-015 (hiPSC, male, UK Erlangen), UKERiRN4-S1-009 (hiPSC, female, UK Erlangen), UKERi4L6-S1-027 (hiPSC, male, UK Erlangen), UKERi33Q-S1-101 (hiPSC, female, UK Erlangen), UKERi82A-S1-002 (hiPSC, female, UK Erlangen)
Authentication	no formal authentication was performed.
Mycoplasma contamination	Regular tested, no contamination was observed
Commonly misidentified lines (See ICLAC register)	No commonly misidentified lines were used.

Plants

Seed stocks	*Report on the source of all seed stocks or other plant material used. If applicable, state the seed stock centre and catalogue number. If plant specimens were collected from the field, describe the collection location, date and sampling procedures.*
Novel plant genotypes	*Describe the methods by which all novel plant genotypes were produced. This includes those generated by transgenic approaches, gene editing, chemical/radiation-based mutagenesis and hybridization. For transgenic lines, describe the transformation method, the number of independent lines analyzed and the generation upon which experiments were performed. For gene-edited lines, describe the editor used, the endogenous sequence targeted for editing, the targeting guide RNA sequence (if applicable) and how the editor was applied.*
Authentication	*Describe any authentication procedures for each seed stock used or novel genotype generated. Describe any experiments used to assess the effect of a mutation and, where applicable, how potential secondary effects (e.g. second site T-DNA insertions, mosiacism, off-target gene editing) were examined.*

Flow Cytometry

Plots

Confirm that:

☐ The axis labels state the marker and fluorochrome used (e.g. CD4-FITC).

☐ The axis scales are clearly visible. Include numbers along axes only for bottom left plot of group (a 'group' is an analysis of identical markers).

☐ All plots are contour plots with outliers or pseudocolor plots.

☒ A numerical value for number of cells or percentage (with statistics) is provided.

Methodology

Sample preparation	For flow cytometry, cells were dissociated using Accutase for 30 mins at 37°C and resuspended in FC buffer (2% FCS, 0.01% sodium azide in PBS). Cells were dispensed into 5 ml tubes (Sarstedt) at 500,000 cells per well. For intracellular antigens, cells were fixed and permeabilized using 100ul BD Fixation/Permeabilization Solution (BD Bioscience) for 10 mins, then 1ml of BD Perm/Wash Buffer was added, cells were incubated for 5 mins and subsequently centrifuged at 1,500 rpm for 3 mins. For intracellular staining of organoid-cells anti-PAX6-APC and anit-MAP2 PE for an additional 30 mins. After a wash step, cells were resuspended in 350µl FACS buffer containing DAPI (1µg/ml). For intracellular staining of neurons, cells were stained using anti-bIII-Tubulin-AF405 (NB600-1018AF405, NovusBio, 1:100) or anti-CTIP2-FITC (ab123449, Abcam, 1:100) for 30 mins. Additional controls included applying an antibody solution without one antibody in the full cocktail ("minus 1 control") that was used to determine potential bleed-through of the fluorophores.
Instrument	Cytoflex S (laser 405nm, 488nm, 561nm, 638nm; Beckman Coulter)
Software	CytExpert 2.4
Cell population abundance	No cells were sorted, only FACS analysis was applied. The abundance of the individual populations are shown in the respective figures.
Gating strategy	Debris was excluded by plotting FSC-A and FSC-Width. Potential singlets were determined in the FSC-A and FSC-H plot. Cells to be analyzed were gated in the FSC-A and SSC-A plot. Gates for antibody- fluorophores for neural/neuronal antigens were determined by using stained and unstained cells as a negative control.

☐ Tick this box to confirm that a figure exemplifying the gating strategy is provided in the Supplementary Information.
